# Hydrogeochemical Response of Cave Drips to Precipitation during Rainfall in a Karst Desertification Region: A Case Study of Shijiangjun Cave, South China

**DOI:** 10.3390/ijerph192315830

**Published:** 2022-11-28

**Authors:** Xiaoxi Lyu, Yuan Li, Kangning Xiong

**Affiliations:** 1 School of Karst Science, Guizhou Normal University, Guiyang 550001, China; 2 State Engineering Technology Institute for Karst Desertification Control, Guiyang 550001, China

**Keywords:** karst rocky desertification, cave drip water, hydrogeochemistry, precipitation, response time, indicators

## Abstract

Exploring the hydrogeochemistry of cave drip water and its response to precipitation events in karst rocky desertification regions is of great significance to the paleoenvironment reconstruction of the karst desertification process using speleothem. We selected three perennial drip sites in the Shijiangjun Cave, located in Guizhou Province, Southwest China, and carried out high-frequency monitoring and sampling during two rainfalls from 22 to 25 May 2016. The major hydrogeochemical parameters of drip water and their relationships with karst desertification were analyzed. The results show that the hydrogeochemistry of the drip water in the Shijiangjun Cave, characterized by HCO_3_-Ca·Mg, was dominated by the dissolution of calcareous dolomite. The three drip sites were classified into the delayed response type (W1) and the rapid response type (W2 and W3) based on the response speed of the drip water indicators to precipitation, which were highly influenced by the piston effect and precipitation dilution, respectively. Furthermore, the response sensitivity of the drip water indicators to precipitation was constrained by the desertification degree in the rainy season, specifically, the faster response appeared in the higher desertification degree area. It is essential to select appropriate drip sites and establish an applicable indicator system for the evolutional history reconstruction of karst desertification using speleothems.

## 1. Introduction

There are large outcropping carbonate rocks and complex three-dimensional geological structures of duality (aboveground and underground) in karst regions, of which the structure soil in the context is distributed in an island-liked pattern [1], resulting in a disproportionate spatial pattern of soil and water resources. The karst eco-environment ultimately has become one of the most typical vulnerable environments all around the world. The vulnerability of the karst eco-environment, as demonstrated by karst rocky desertification, has restricted local social and economic development in the karst region of South China [2,3]. In recent years, many innovative methods have been utilized to monitor environmental changes before and after karst rocky desertification control, including remotely sensed imagery interpretation, the dynamic variations of vegetation and soil physicochemical properties, and so on [3,4,5,6,7,8]. Although the above surface monitoring methods provide promising approaches to the establishment of a karst rocky desertification monitoring system, however, the research on the recent conditions and variations of karst rocky desertification by way of underground medium or indicators has been rarely reported until now. Therefore, building an integrated environmental monitoring system based on the characteristics of dual structure in karst regions is extremely important for karst rocky desertification control evaluation and would be a breakthrough in improving and developing the current monitoring system.

Speleothems in karst caves (stalagmites) are formed due to the interactions of a multiple-surface environment, such as precipitation, an overlying ecosystem, karst seepage water, and other systems [9,10], and they record abundant information on the paleoclimate and paleoenvironment [11,12,13]. Coplen et al. previously analyzed the stalagmite δ^18^O and δ^13^C in the Devils Hole in Nevada and reconstructed the paleoclimate and paleovegetation changes in the past 500,000 years in this region [14]. A consensus has been reached that the stalagmite δ^13^C obviously responds to the CO_2_ δ^13^C in the soil overlying the carbonate rock layer, and subsequent studies have focused on using the stalagmite δ^13^C to trace the changes in the paleoenvironment and the recent eco-environment [15,16,17]. Liu et al. compared the stalagmite δ^13^C in Guizhou Province, Southwest China, and found that the stalagmite δ^13^C in the Shijiangjun Cave was close to that of the surrounding bedrock and far higher than that in the Zhijin Cave [18], the evolutional history of karst rocky desertification in the past 800 years was further discussed in terms of the stalagmites δ^13^C and δ^18^O in these two caves, which proved the influence of karst rocky desertification degree on stalagmites δ^13^C. Cave drip water is an important medium of speleothems formation, which exhibits different geochemical characteristics in various karst rocky desertification environments. For instance, drip water that developed in severe karst rocky desertification conditions was commonly characterized by lower Ca^2+^ and Mg^2+^ concentrations than that in a mild desertification environment, as well as variable amplitudes in their ionic concentrations [19]. Although previous studies have presented valuable results, there are still obvious differences in the seepage paths and hydrodynamic properties in the formation process of drip water, hydrochemical compositions of drip water, as it described via the indicators, reflects the constraint by various kinds of hydrogeochemical processes [20], which will certainly influence the response degree of drip water indicators to the overlying environment of karst rocky desertification, and lead to various interpretations in revealing environmental changes by drip water. Therefore, it is critical to systematically sort out and deepen the existing work.

Generally, the signal transmission of groundwater from the earth’s surface to the cave is highly influenced by vegetation type, soil depth, bedrock thickness, cave pattern, drip water location, and hydrological and meteorological conditions [21,22]. However, due to the unique binary karst geological structure, the response of groundwater to atmospheric precipitation is relatively more sensitive than that in non-karst regions [3]. The investigation into hydrogeochemical characteristics of cave drip water under the condition of precipitation is not only conducive to the identifications of hydrological processes (recharge, storage, and migration) of seepage water in the upper vadose zone [23,24], but is also significant to the environment signal interpretation on the basis of speleothems for the reason that the formation process and influencing factors of cave speleothems could be better understood [25]. The drip sites of the Shihua Cave in north Beijing, China, were classified into two groups (rapid and delayed response type) according to the response characteristics of the drip water to precipitation [26]. The response of the drip water to precipitation in the Grotta di Ernesto Cave in northeast Italy was characterized by three patterns: rapid seasonal drip water, slow seasonal drip water and slow drip water [27]. Research on the Liangfeng Cave in Southwest China revealed that information on extreme precipitation events could be recorded by the hydrochemistry and stable isotope variations of drip water, and the intensity of precipitation played a key role in constraining the Ca^2+^ and HCO_3_^−^ concentrations [28]. Owing to the complexity of hydrological processes from the soil layer to the surface vadose zone, it is difficult to fully understand the historical information of each drip site. However, the frequency and contemporary hydrogeochemistry of drip water under precipitation conditions provide some clues to clarify the hydrological processes of karst groundwater, such as the water-rock interaction degree, the water retention time, the piston effect, and so on [29]. Therefore, the Shijiangjun Cave under the conditions of moderate karst rocky desertification was selected as the study area. Three drip sites were monitored and sampled at high frequency during the rainy season, aiming to interpret the particular response characteristics of drip water hydrogeochemistry to precipitation in typical karst rocky desertification areas and to explore which indicators of drip water under different hydrologic conditions could potentially reflect the surface environment. This research is expected to provide theoretical references to the paleoenvironment reconstruction of karst rocky desertification using speleothem and karst rocky desertification control at the current stage.

## 2. Materials and Methods

### 2.1. Study Area

**Figure 1:**
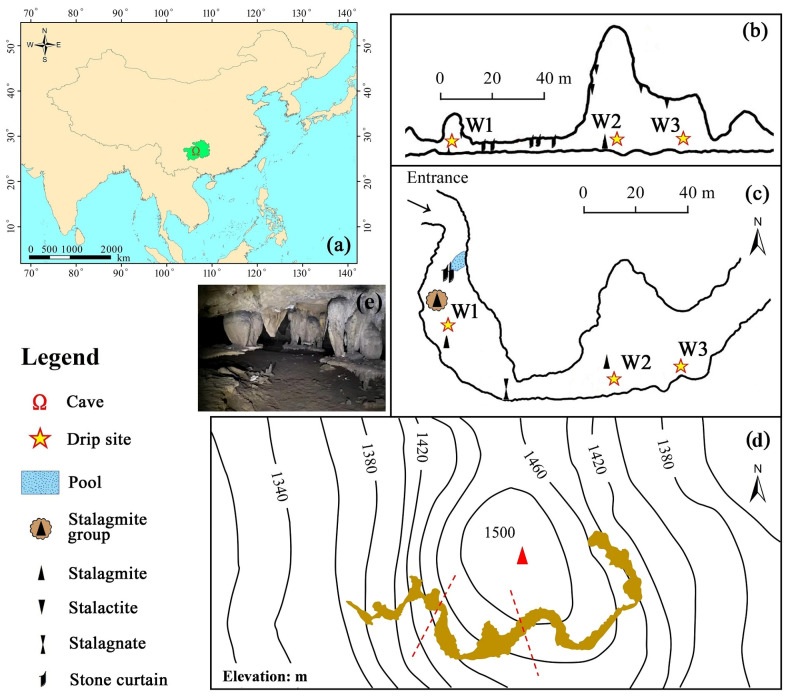
The selected drip sites in the Shijiangjun Cave (**a**): location of the Shijiangjun Cave in China; (**b**): longitudinal profile of cave sampling section; (**c**): planar graph of the sampling section in cave passage; (**d**): topographic map of the surrounding area, the dark-yellow region represents the planar graph of the Shijiangjun Cave, and the red dotted lines represent the cave sampling section; (**e**): speleothems and sediments in the Shijiangjun Cave.

The Shijiangjun Cave (26°17′03′′ N, 106°03′54′′ E) is located in the central part of the Guizhou Karst Plateau, Southwest China (Figure 1a), which is the watershed between the Yangtze River Basin and the Pearl River Basin. It developed in the thick Middle-Upper Triassic calcareous dolomite strata (T_2p-l_) with a small amount of evaporative minerals (mainly halite), sandstones and shales in the interlayers [30]. Tectonically, it is located at the southwestern edge of the Sichuan-Guizhou Meridional Structural System, and it developed in the west wing of the Maolipo Anticline, the formation dips are generally from 10° to 25° [30]. The area around the cave system is characterized by a typical subtropical monsoon climate, with an average annual precipitation of 1290~1380 mm and annual temperature of 14~16 °C, and the rainy season is usually from May to October. The geomorphic assembled forms are mainly fengcong (peak cluster)-valley and fenglin (peak forest)-depression, and poljes are quite common for this karst plateau. The regional base level of erosion is close to the bottom of the depression, and the groundwater type is mainly carbonate fissure-cavity water. Shrub grass is the main vegetation type in the surrounding region, with most of it normally distributed in the low-lying areas of mountains. Moderate and intense karst rocky desertification developed in the peak body, and the karst rocky desertification in the overlying environment of the Shijiangjun Cave was graded as moderate due to the steep peak body and the high rock exposure rate of more than 50%.

The Shijiangjun Cave, which is more than 500 m in length was formed in the Shanpen Period (a physiographic stage from the Early Miocene to the Pleistocene), during which period the earth’s crust was stable and most cave channels were horizontally developed [30]. The only cave entrance appears at the bottom of the peak body on the edge of the depression (Figure 1d), and the cave halls are distributed in a beaded-like form. The cave width is from 3.1 to 48 m and the cave height is from 1.8 to 20.3 m. The rock thickness over the cave ceiling is from 79.7 to 118.2 m. Many vertical fissures have developed in the cave roof, including tufted stalactites, sodastraws, and stone curtains due to the chemical precipitation effect of drip water. There are abundant drip sites and well-developed speleothems, such as stalagmites, sodastraws, stalactites, stalagnates, stone curtains, stone flags, and so on. The drip water forms several discontinuous water flows in the cave passage and flows underground. The average annual CO_2_ concentration in the cave is 409 ppm, the annual temperature is 15.16 °C, and the annual humidity is 93%. There are small amounts of shrubs and grasses growing in the peak body, and only a few small trees growing in deep soil layers and large fissures, however, there are many bare rocks and thinner soil layers overlying the cave. Most soils were washed into the cave space through the cavernous fissures and cracks in the past, forming a 2~4 m sedimentary layer at the bottom of the cave tunnel, and most huge stalagmites developed on the humid sediments (Figure 1e).

Three perennial drip sites, numbered sequentially as W1, W2, and W3 and located in the middle section of the cave tunnel (Figure 1b,c), were selected as the monitoring objects in this study, the drop heights of which are 7.1, 20.1, and 13.1 m, respectively. The cave roofs at the three drip sites consist of calcium plate and new carbonate deposits, and several stalagmites have developed below each drip site.

### 2.2. Field Monitoring and Sampling

Monitoring and sampling were conducted at the three drip sites every 4 h from 23:00, 22 May to 20:00, 25 May 2016 after a long dry period and before the following rainstorm, and only 54 samples were obtained. Real-time temperature and rainfall data were collected using the WM5200 weather station (Vaisala Company, Helsinki, Finland) in the open space near the cave. The drip speed of the drip water was measured by a stopwatch in each sampling group, and the average drip volume, defined as the drip rate in this study (unit: mL·min^−1^), was calculated by measuring the water quantity with a 100 mL glass cylinder in 5 min. The water temperature, pH, and electrical conductivity (EC) of the water samples were determined in situ by a portable water quality analyzer (HQ40d, HACH Company, Loveland, CO, USA) with the measurement precisions of 0.1 °C, 0.01 and 1 µS·cm^−1^, respectively. The pH probe was calibrated with buffer solutions with pH value of 4.005, 7.000, and 10.012 before the measurement. The HCO_3_^−^ concentration was determined by a basicity meter (Merck Company, Darmstadt, Germany) with the test precision of 0.1 mmol·L^−1^. Water samples were collected in a 50 mL fully rinsed polyethylene bottle, meanwhile, 1:1 HNO_3_ was added to acidify the sample to pH < 2 in order to ensure the activity of the cations. Then, the polyethylene bottle was sealed with parafilm (Bemis Company, Neenah, WI, USA) for the subsequent determination of the cations. The remaining water samples were put into a 250 mL sampling bottle. Then, all samples were placed in a portable refrigerator in the field and refrigerated at 4 °C. Finally, the water samples were brought back to the laboratory for cryopreservation, and the cations and anions were tested within 48 h. All the above-mentioned sampling bottles were soaked in dilute acid for 24 h before sampling, and then washed with deionized water more than three times.

### 2.3. Laboratory Measurements

The samples of drip water were brought to the Geochemical and Isotope Laboratory of Southwest University, China, to detect the cation concentrations of K^+^, Na^+^, Ca^2+^, Mg^2+^, and Sr^2+^ by using an Optima-2100DV inductively coupled plasma emission spectrometer (ICP-OES, Perkin-Elmer Company, Waltham, MA, USA). The detection precision was 0.001 mg·L^−1^ with a standard deviation of less than 2%. NO_3_^−^ and SO_4_^2−^ were analyzed by a UV2450 ultraviolet spectrophotometer produced by the Shimadzu Company (Kyoto, Japan), and the Cl^−^ was tested according to the method of AgNO_3_ titration. All samples were filtered by glass fiber membranes (Whatman Company, Maidstone, UK, 0.45 μm) before the ion determination. In addition, the hydrogeochemical simulation program of WATSPEC 15 (Wigley, Adelaide, Australia) was used to calculate the calcite saturation index (SI_C_) and dolomite saturation index (SI_D_) of the water samples based on the data sets of the water temperature, pH, and main ions. The data statistics and analyses were completed in IBM SPSS Statistics 20, and cartographic drawing was performed using Origin 2018 software.

## 3. Results

### 3.1. Physico-Chemical Characterization of Cave Drip Water

The comparative analysis of the drip water hydrological and hydrochemical parameters during the precipitation process (Table 1) at the three drip sites in the Shijiangjun Cave shows that the drip rate in unit time at each drip site displays a relationship of “W2 < W1 < W3” (1.79 ± 0.25, 2.01 ± 0.12, 2.22 ± 0.31 mL/min, respectively). The drip rate and the variable amplitude had the largest values at W3, and the hydrological conditions of W2 were comparatively more stable during the monitoring period. All the drip water samples presented obvious weak alkalinity, while both W2 and W3 exhibited slightly stronger alkalinity than that of W1 (8.62 ± 0.14). The EC shows a relationship of “W1 > W2 > W3”, and the range of variation in W1 was much larger than that of W2 and W3 (364.56 ± 21.66, 272.61 ± 1.54, 231.39 ± 0.78 μS/cm, respectively). Most cation and anion concentrations had a consistent variation with the EC except for those of K^+^, Sr^2+^ and HCO_3_^−^. The variable amplitudes of most ion concentrations at W1 were higher than those of W2 and W3, showing a similar spatial pattern of EC. Generally, Ca^2+^ and Mg^2+^ dominated the cations in the samples collected from the three drip sites, while HCO_3_^−^ dominated the anions, showing the typical hydrochemical characteristics of karst regions.

The dataset collected at each drip site was processed in the WATSPEC 15 program (Table 1). The results show that both the SI_C_ and SI_D_ for the drip water were greater than zero, showing a supersaturated status of calcite and dolomite in the drip water. For the three drip sites, the mean values of SI_D_ (1.68 ± 0.23, 1.55 ± 0.12 and 1.49 ± 0.11, respectively) were significantly larger than those of SI_C_ (0.89 ± 0.11, 0.72 ± 0.06 and 0.63 ± 0.66, respectively), which indicates that the calcite precipitation effect in drip water was weaker than that of dolomite and the dolomite had a stronger precipitation tendency than that of calcite. This also reflects that the drip water hydrochemistry mainly inherited the characteristics of dolomite [31], which is consistent with the geological background of calcareous dolomite in the Shijiangjun Cave. Furthermore, all the carbonate saturation indices, including the SI_C_ and SI_D_ at the three drip sites follow a rank order of “W1 > W2 > W3”, indicating that there was a relatively better dissolution ability of the drip water of W2 and W3 than that of W1; in other words, W1 was more prone to calcite and dolomite precipitation than W2 and W3. 

### 3.2. Proportions of Cations and Anions

**Figure 2:**
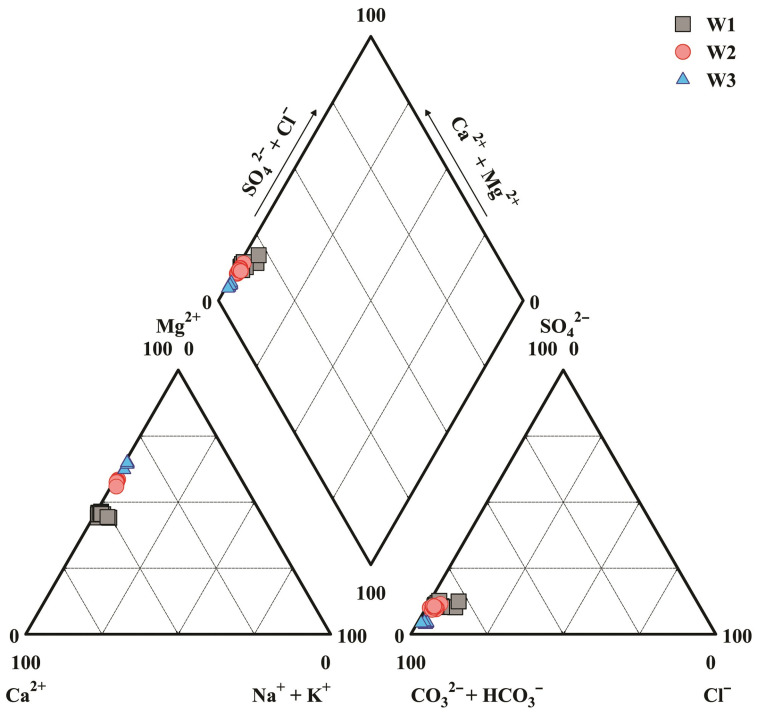
Piper diagram of drip water hydrochemistry.

The drip water hydrogeochemical characteristics can also be seen in the following Piper diagram (Figure 2). The proportions of each cation to the total cations were significantly different at the various drip sites. At W1 and W2, the proportions of Ca^2+^ (57.85~65.21% and 52.22~53.9%) were obviously greater than those of Mg^2+^ (30.6~34% and 43.19~45.93%). On the contrary, a relatively higher proportion went to Mg^2+^ at W3 (49.7~52.99%). It can also be seen that, the proportions of K^+^ for W2 and W3 (0.43~0.73% and 0.56~0.93%) were both lower than those of Na^+^ (1.19~2.31% and 0.81~1.6%), while the average K^+^ proportion of W1 (2.44%) was more than twice that of Na^+^ (1.1%), and the proportion of Sr^2+^ to total cations was the lowest at all drip sites (0.03%, 0.02%, and 0.02%). In addition, the proportions of anion concentration to the total anions at all three drip sites are basically ranked as “HCO_3_^−^ > SO_4_^2−^ > Cl^−^ > NO_3_^−^”. Specifically, the anion proportions were 87.33%, 8.96%, 2.37%, and 1.34% for W1, 88.92%, 8.13%, 1.54%, and 1.42% for W2, 94.21%, 3.21%, 1.58%, and 0.99% for W3.

The hydrochemical types of drip water could be classified into the type of HCO_3_-Ca·Mg based on the analysis results of the Piper diagram, which also shows the particular hydrochemical composition in the dolomite region. The concentrations of Na^+^ and K^+^ were relatively lower, which indicates that the silicate weathering contributed limitedly to the cations. The above fact suggests that carbonate weathering plays a dominant role in the hydrochemical composition of drip water, especially the dolomite dissolution. The small amount of Na^+^ and Cl^−^ in the drip water is derived from the evaporite minerals dissolution in the dolomite strata [32]. Comparatively, the rainwater had a limited contribution to the substances in the drip water. According to the previously investigated data (Table 2), the max ion concentrations of the rainwater were relatively lower, for the cations (K^+^, Na^+^, Ca^2+^, Mg^2+^, and Sr^2+^), the max values were 0.37, 0.2, 2.8, 0.63 and 0.003 mg·L^−1^, respectively; and for the anions (HCO_3_^−^, SO_4_^2−^, NO_3_^−^, and Cl^−^), the max values were 12.2, 1.48, 1.01, and 0.38 mg·L^−1^, respectively. Rainwater mainly affects drip water formation by changing the seepage path and hydrodynamic conditions [33].

## 4. Discussion

### 4.1. Precipitation Response and Hydrological Process

According to the data statistics from a small meteorological station, there were two precipitation events in this monitoring period. The first precipitation was from 23:07, 22 May to 14:20, 23 May 2016, and the second was from 0:11, 24 May to 15:42, 24 May 2016. The rainfall amounts were 24.7 and 8.3 mm with the peak values appearing at 7:00, 23 May and 6:00, 24 May, respectively.

**Figure 3:**
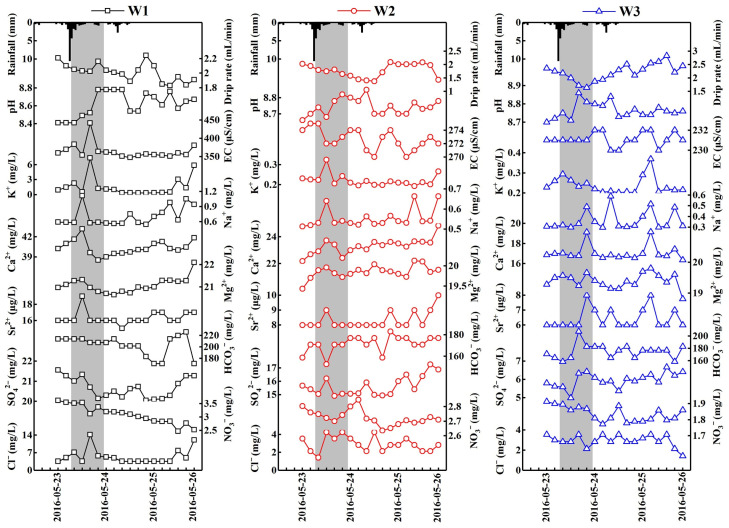
Variations of hydrogeochemical characteristics and the responses of drip rate to precipitation for three drip sites during precipitation events.

Based on the analysis of the relationship between the drip rate and the rainfall, it was found that the drip rate at W1 showed a downward trend during the first precipitation event, and a transient increase at 20:00 on 25 May (Figure 3). It began to rise 4 h after the rainfall peak in the second precipitation event, and a peak value appeared after about 10 h, then declined, and a valley value appeared until 8:00 on 25 May. The drip rate at W2 showed a declining tendency during the previous precipitation, gradually increased in the second precipitation, and then declined after maintaining a stable state for 16 h, and reached its peak around about 20:00 on 24 May. The drip rate at W3 also showed a downward trend after the initial precipitation and increased obviously at 20:00 on 23 May. The first peak value appeared after the second precipitation, and the second one appears at 20:00 on 25 May. Thus, it can be preliminarily concluded that the W1 had the largest hysteresis in responding to the precipitation, while the W2 and W3 were sensitive to respond to precipitation and showed better seepage path connectivity. Both W2 and W3 were classified into the drip site group of the rapid response type.

By comparing the variation trend of the EC and pH in different precipitation periods (Figure 3), we further confirmed the above understanding. The peak value of the EC for W1 appeared 8 h after the beginning of the first precipitation, and the EC started to gradually increase after 2 days of a stable period. It is inferred that there is an overlying thick cave roof above the site of W1. As a result of the influence of the “piston effect”, the “old water”, which was stored in the epikarst before the precipitation [34,35], pioneered to enter the seepage path and formed drip water with a high value of EC. Subsequently, the “old water” flowed into the underground cavity, and the EC of drip water became to stabilize, and started responding to the precipitation in the exoteric environment until the increase in the EC in the later period. The EC of both W2 and W3 exhibited intense fluctuations, declining at least three times during the precipitation period, but the average EC of both sites was lower than that of W1. The variation in the pH values shows a rising tendency after the precipitation, which was relatively lagging behind that of the EC. According to the results of a previous study, the karst groundwater hydrogeochemistry in the process of precipitation is sensitive to the dilution of rainwater [36], and the EC decreased along with the increasing drip rate at W2 and W3, demonstrating that the drip waters at both W2 and W3 in the Shijiangjun Cave were also significantly affected by the dilution effect. In addition, this also indicates that the retention time of the seepage water in strata was relatively shorter, and the water rock interaction was insufficient, suggesting better fissure connectivity above the two drip sites. Therefore, the latter two drip sites responded rapidly to the precipitation events, further confirming the above judgment.

**Figure 4:**
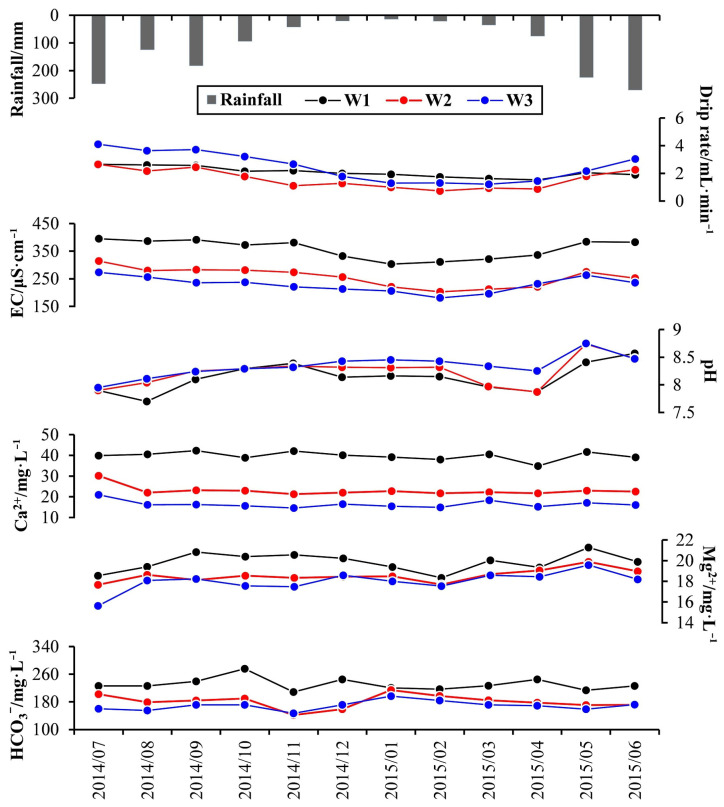
Time series of major hydrologic and hydrogeochemical characteristics for cave drip water in the Shijiangjun Cave (2014~2015).

**Figure 5:**
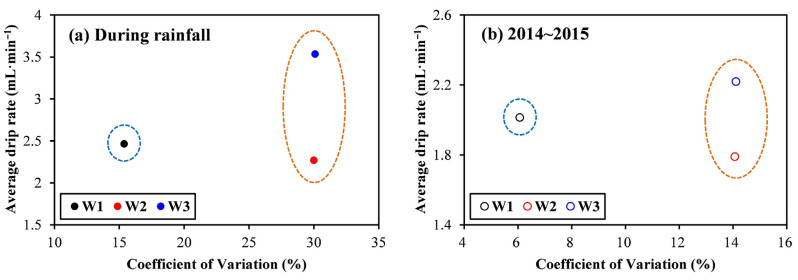
Comparisons between drip rates for the three selected drip sites during this rainfall monitoring (**a**) and the hydrological year from 2014 to 2015 (**b**).

The annual and high-resolution monitoring of drip rate, EC, and other parameters of drip water helps to clearly explain the seasonal variations of drip water and aquifer structure of the karst cave [37]. Therefore, we compiled a data graph set on the physical and main ion parameters of the drip water from July 2014 to June 2015 in the Shijiangjun Cave (Figure 4). The data atlas includes the average monthly rainfall, the drip rate (in mL/min), EC, pH, calcium, magnesium, and bicarbonate of the monthly water samples of the three drip sites. The field sampling of monthly water sample was completed at the end of each month. It is showed that all the drip rates and EC of the three drip sites had positive responses to the precipitation in the monitoring of this hydrological year. Overall, the drip rates and EC of the three drips were at a high level in the rainy season, while relatively lower in the dry season (from November 2014 to April 2015). In comparison, there was a relatively stable trend of the seasonal variation of drip rates and EC for W1, while those of W2 and W3 fluctuated greatly, especially for W3. According to the relationship between the average drip rate and the Coefficient of Variation of the drip rate, the three drip sites can be clearly classified into two groups during both the rainfall monitoring (Figure 5a) and the hydrological year (Figure 5b). The first type includes W2 and W3 with large variable amplitudes of drip rate and obvious response to rainfall, and the other one only includes W1, which had relatively stable variation of the drip rate. Furthermore, the drip rates and EC of W2 and W3 obviously decreased with the rainfall in August 2014, while there was not significant change for W1. The drip rates and EC of the three drip sites showed obvious upward trend with the increasing rainfall after a long dry period in May 2015. Specifically, the variable amplitude of EC for W1 was much larger than those of W2 and W3, which again confirmed the above viewpoint that W1 was significantly affected by the piston effect. Although the EC of W2 and W3 also increased, the overall level (about 250 μS/cm) was much lower than that of W1 (350 μS/cm), which was related to the abovementioned rainwater dilution and insufficient water rock interaction, indicating that the karst tunnels in aquifers above W2 and W3 had stronger connectivity than that of W1.

### 4.2. Response of Drip Water Geochemistry to Precipitation

The results of the tracer experiment using NaCl, related to the hydrodynamic process of cave drip water responding to precipitation [38], suggest that the drip water in the Shijiangjun Cave responded to the atmospheric precipitation in a timely manner and indicate that the rainwater could quickly pass through the strata in the cave roof, so that the retention time of underground water was shortened and the water rock interaction intensity was weakened owing to the vertical fissures in the cave roof. Then, a substantial number of the main chemical compositions of the drip water were directly derived from the eluviation of the overlying soil layer. As reported in previous research, Ca^2+^ and Mg^2+^ in the drip water of the Shijiangjun Cave were restricted by the development degree of the overlying soil [20]. Specifically, the Ca^2+^ and Mg^2+^ concentrations in the drip water increased with the thickness of the soil layer; in other words, the thicker soil layer contributed more Ca^2+^ and Mg^2+^ to the drip water. As the dominant cations of the drip water, the Ca^2+^ and Mg^2+^ variations were essentially in accordance with that of the EC from the monitoring data at the three drip sites during the rainfall process in this study. The Ca^2+^ and Mg^2+^ concentrations at W1 gradually stabilized after a brief fluctuation, then presented a slight upward trend, indicating that the surrounding rocks were fully dissolved by the groundwater with the extension of the water retention time, so that the response of the drip water to the precipitation was relatively more delayed. The Ca^2+^ and Mg^2+^ concentrations at W2 and W3 fluctuated greatly, with a rapid increase after each precipitation (Figure 3), indicating that the drip water was mainly “new water” formed in the shallow soil layer after each precipitation event and that the Ca and Mg in the soil layer were quite vulnerable to be leached and lost by rainwater [39].

The seasonal variations of Ca^2+^ at the three drip sites were relatively stable in the monthly monitoring of a hydrological year, there were not obvious peak and valley values of calcium ions during the observation (Figure 4), which is different from those of the major ions at the perennial drip sites of other caves in South China. For example, the high-frequency study of drip water during rainfall at six drip sites in the Liangfeng Cave, Guilin, showed that Ca^2+^ had a large variation (40~120 mg/L) in the dry season from October, 2015 to January, 2016 [28], it was pointed out that the rainfall in the dry season was the major factor affecting the hydrochemistry of drip water. However, the Ca^2+^ concentrations of the drip water from the Shijiangjun Cave had remained at a relatively lower level throughout the hydrological year, which may be affected by the prior calcite precipitation. In contrast, the seasonal fluctuations of Mg^2+^ concentrations were significantly larger than those of Ca^2+^, and the variation trends were close to those of pH in rainy season, which indicates that the high temperature and groundwater full of CO_2_ promoted the dissolution of calcium dolomites in rainy season.

**Figure 6:**
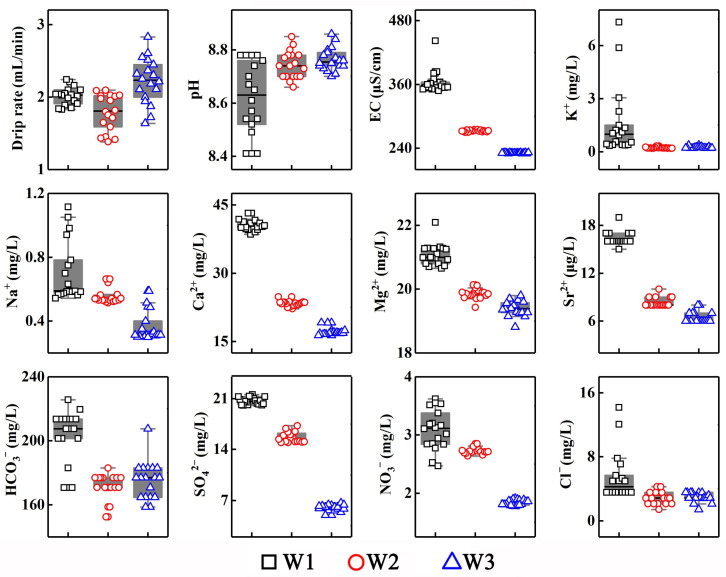
Spatial pattern of drip water hydrogeochemical indicators in the Shijiangjun Cave.

Generally, K^+^ and Na^+^ in natural surface water are mainly from atmospheric precipitation, silicate weathering, and evaporite dissolution. However, those in the cave drip water are largely contributed by the input of soil layer [40]. Accordingly, because the bedrock in the Shijiangjun Cave is mainly composed of calcareous dolomite and the surrounding environment is hardly influenced by human activities, the K^+^ in the drip water is mainly derived from the eluviation of the overlying soil, while the atmospheric precipitation contributes less to the K^+^ concentration (see Section 3.2). In addition to the dissolution of halite, soil leaching is also one of the sources of Na^+^. Since the K^+^ and Na^+^ concentrations at the three drip sites were relatively lower and influenced by the dilution effect of rainwater to various degrees, their responses of to the variations in rainfall are not obvious. Typically, clay minerals in the soil layer have strong adsorption properties to the element of Sr. It was pointed out that the Sr^2+^ in the drip water was relatively active, and its concentration variation was closely related to the precipitation outside in previous studies [41], which could also be found in the fluctuations of the Sr^2+^ concentration at W2 and W3 with the precipitation events and is consistent with the results of our study. Furthermore, the spatial pattern of the hydrogeochemical indicators shows that most of the cation concentrations at W1 were higher than those at W2 and W3 (Figure 6), indicating that the response sensitivity of the drip water indicators to precipitation was influenced by the differences in the material sources for the various drip waters.

**Figure 7:**
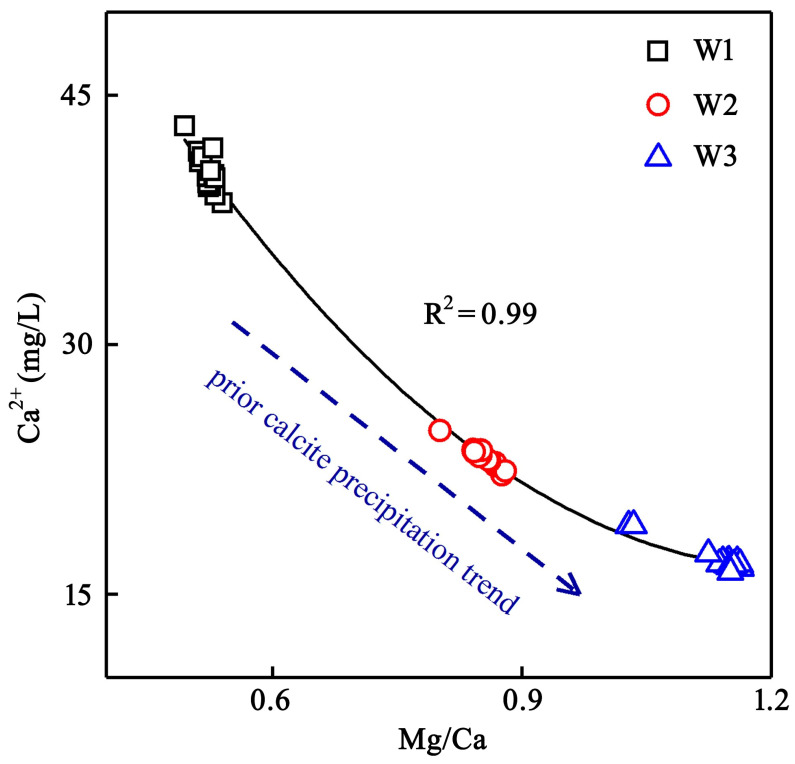
Correlation between Ca^2+^ and Mg/Ca at the three selected cave drip water sites.

As one of the main participants in karstification, bicarbonate occupies a large proportion of the total anions and TDS at the three drip sites. However, the dynamic changes in the HCO_3_^−^ were not consistent with that of the EC during the precipitation, especially at the drip site of W1. The HCO_3_^−^ of W1 showed a better steady behavior during the first precipitation, as suspected from the previous studies [34,38], and there was a thick soil layer above the drip site of W1. It started to decline gradually in the second precipitation during the first several hours, then increased until 4:00, 25 May and decreased again 12 h later. The rainwater, flushing through the soil layer in the karst region, carries a great amount of CO_2_ and flows into the karst fissures or karst tunnels, forming drip water with supersaturated carbonate. Actually, this resulted in a decreased trend of Ca^2+^ with the increasing Mg/Ca, indicating that the calcite saturation and precipitation had occurred before the seepage water drops (prior calcite precipitation) (Figure 7), which could also be verified by the carbonate suture structures distributed in the cave roof, especially for W2 and W3. Meanwhile, the fissures developing in the cave roof strengthened the vertical connectivity of the strata, resulting in carbonate-saturated water prone to CO_2_ degassing, which led to the effect of prior calcite precipitation effect. Furthermore, both the SI_C_ and SI_D_ of W1 were generally large compared to those of W2 and W3 (Table 1), and in Figure 7, the data points of Ca^2+^ vs. Mg/Ca for W1 tended to be closer to the weak end of prior calcite saturation than those of W2 and W3, which can be summarized as follows: the W1 could be characterized by different hydrogeochemical processes compared to W2 and W3. 

The geochemical behavior of chlorine, which is mainly from sea salt deposition, is relatively conservative in the natural process of the water cycle, therefore, Cl^−^ is usually used as a potential indicator and tracer to explore the response of drip water to atmospheric precipitation [42]. Although Cl^−^ is not the main component of anions in the drip water in the Shijiangjun Cave, it had a significant response to both of the two precipitation events due to the characteristics of a single source, demonstrating the dilution effect of rainwater on the hydrogeochemistry of drip water (Figure 3). Particularly, there were two peak values of Cl^−^ for W1 during the monitoring period, indicating the response of drip water to precipitation influenced by the piston effect, while the following lower values after each peak shows the impact of rainwater dilution. It is worth mentioning that the response degrees of different drip sites are subject to the thickness of the cave roof and multiplicate flow paths. The variation trends of SO_4_^2−^ and NO_3_^−^ in drip water were not consistent throughout the precipitation process in this study. The SO_4_^2−^ concentration is characterized by an intense fluctuation with a slightly increasing trend and shows a better response to atmospheric precipitation. Previous studies have pointed out that the sulfur element in drip water is not directly derived from the atmosphere, but contributed by the sulfur compounds (the coal seam or coal soot) in the cave roof [43,44]. The overlying soil environment is another source of sulfate in cave drip water. Specifically, the sulfur fixed in the soil layer is oxidized and forms sulfate before the precipitation [45]. The leaching effect of the rain increases the SO_4_^2−^ concentration and maintains a high level until the preferential flow disappears. The changes in nitrate in drip water in the Shijiangjun Cave were obviously different from that of sulfate. The NO_3_^−^ concentration of W1 presents an obvious downward trend with fewer fluctuations, while those of W2 and W3 reached a peak after the first precipitation, and gradually increased after the second one. Generally, the NO_3_^−^ concentration followed the rank order of “W1 > W2 > W3” at the different drip sites from an overall perspective (Figure 6) due to the better soil development at W1. The nitrate in the drip water was mainly derived from the overlying soil nitrification. Therefore, the changes in the NO_3_^−^ in the drip water mainly reflect the different leaching degrees of the soil under the condition of precipitation.

### 4.3. Influence of Karst Rocky Desertification on the Response of Drip Water Indicators to Precipitation

The classification standard list of the degree of karst rocky desertification involves multiple parameters, such as the vegetation coverage, the rate of outcrop rock, soil depth, slope, and so on [2]. As a result, the karst rocky desertification grade is actually used as a comprehensive evaluation of the karst rocky desertification degree at the regional scale. Furthermore, the karst region itself is characterized by discontinuous vegetation and soil layer, and the heterogeneity of the eco-environment displayed more obviously in moderate and intense karst rocky desertification regions. In particular, the strong aggregation effect of the bare rocks not only leads to an inhomogeneous intra-regional distribution of soil and vegetation, but also results in the differences in the soil physicochemical properties and the plant species diversity [46]. Accordingly, the numerous drip water developed in the same cave experienced different overlying environments in the formation processes, and the surface karst rocky desertification degree above each drip site is different. As mentioned above, the overlying bedrocks for W2 and W3 are relatively thinner, with developed fissures and well-connected groundwater channels; therefore, the drip waters carry more environmental information about karst rocky desertification driven by rainwater. For instance, the cations of K^+^ and Sr^2+^ in the drip water can better reflect the leaching degree of the overlying soil, as well as the mineral nutrients in the soil under the background of karst rocky desertification to some extent. However, for W1, the elevation of the drip site is relatively lower than those of W2 and W3, the overlying soil layer and strata are thicker, with a milder karst rocky desertification degree, and the flow paths are of poor connectivity. Therefore, this reflects the combined effect of the karst rocky desertification environment and bedrocks on the dynamic variation in the indicators of cave drip water [34,38], which results in a slightly lagging response to the precipitation event, as shown by the indicators. Especially, the Ca^2+^ and Mg^2+^ are also contributed by the detained water with full water rock interaction in the karst strata, from which the “piston effect” resulted in the less sensitive response of those hydrogeochemical indicators to karst rocky desertification and seriously interfered with the accuracy of the indicators reflecting the external eco-environment.

In order to further clarify the effect of the karst rocky desertification environment on the response of drip water indicators to atmospheric precipitation, the response time of the drip water in the rainy season for the Shijiangjun Cave was investigated and compared with those of other drip waters developed in different karst rocky desertification environments (Table 3). It is necessary to state that all the selected karst caves are located in the subtropical environment in South China to ensure a scientific and effective comparison, and the grade of karst rocky desertification overlying each cave is summarized according to the original description in the reference literature, as well as the response time data collection. The response time of drip water to the external atmospheric precipitation decreases with increases in the karst rocky desertification grade. Specifically, the drip water developed in the moderate-intense grade of karst rocky desertification generally responds to precipitation events rapidly within 1~2 days. Nevertheless, the drip water developed in the non-desertification and mild desertification regions requires at least a month to respond to precipitation events. The above-mentioned difference is mainly considered to be due to the overlying soil [38]. In general, the perennial type of drip water is principally supplied by atmospheric precipitation during the rainy season [24]. Comparatively, the soil layer developed in the moderate-intense grade of karst rocky desertification is thinner, with a poorer capacity for water conservation, such as the Xiaoyan Cave, Guangxi, South China. The soil structure is vulnerable and easily destroyed by the splash erosion of raindrops. As a result, the rainwater can quickly flow into the karst fissures and tunnels through the overlying soil layer. Furthermore, the carbonate rocks located in regions with intense degrees of karst rocky desertification are also characterized by a high level of dissolution and developed fissures [34], which leads to a relatively rapid response of the drip water to precipitation events.

### 4.4. Implications for the Paleoenvironment Reconstruction of Karst Rocky Desertification Using Speleothem

During the formation of drip water, the rainwater carries a large amount of calcium, magnesium minerals, and organic matter when it flows through the soil surface and into the underground space (i.e., pores, fissures, or caves). As a result, the formation of cave drip water is also a process of nutrient leakage and loss in karst regions. The soil and water leakage causes the loss of surface water and soil resources, leading to the damage of vegetation living conditions, a more fragile karst ecosystem, and the aggravation of karst rocky desertification [49]. Furthermore, the karst rocky desertification regions have unstable soil structure and an intense infiltration capacity of rainwater, in which the seepage water fills the karst fissures and dissolves the bedrocks, accelerating the development of fissures and pipelines, and transporting soil particles and nutrients to the underground space. Ultimately, it is inevitable that ecological degradation forms a vicious cycle in karst rocky desertification regions.

The surrounding environment of the Shijiangjun Cave is characterized by the steep slope of the peak body, high exposure rate of overlying rocks, a lower vegetation coverage rate and thinner soil layers. The fissures and karrens are extremely developed, which results in the strong connectivity of the flow paths and the main flow types of preferential flow and matrix flow [38] so the hydrogeochemical parameters of drip water rapidly respond to the external atmospheric precipitation. The retention time of moisture is relatively short and the water rock interaction is relatively weak in the strata due to the vertically developed fissure structures. Some geochemical indicators, such as SO_4_^2−^ and NO_3_^−^, inherit the signals of the overlying environment directly. The results from a previous investigation indicated that the SO_4_^2−^ in drip water could be used as a tracer to indicate the surface biomass [43,45,50], which provides possibility and a reference for the reconstruction of the paleoenvironment using speleothems in the karst rocky desertification areas. However, the contents of cations (Ca^2+^, Mg^2+^ and Sr^2+^) in drip water coming from bedrock dissolution are inevitably and seriously affected by many hydrogeochemical factors, such as dilution effect, water sources, and prior calcite precipitation. The information carried by the cations in drip water may help researchers to understand the evolution process of karst rocky desertification. Therefore, they can explain the surface environment status and evolution process of karst rocky desertification on the premise of tracer mass sources so as to avoid the uncertainty generated from the interpretation of the overlying environment through the analysis of the hydrogeochemical indicators.

Some researchers have paid attention to the scientific values of organic indicators in their research on paleoclimate and paleoenvironment by stalagmites [51,52,53,54]. The organic matter in cave drip water is mainly derived from the overlying soil and vegetation, which maintain stable properties during the migration of seepage water. The signals carried by organic matter can effectively conserve the environmental information of overlying vegetation and soil. This preferably avoids the uncertainty in the response to climatic or environmental changes by using the stable isotopic composition of inorganic carbon and has the potential advantages of recording the changes in the climate and surface environment, as well as the important reference values in reconstructing the formation and evolutional history of karst rocky desertification using speleothems. Furthermore, the selection of appropriate drip sites is also of great importance for the speleothem−based paleoenvironment reconstruction of karst rocky desertification, in addition to the establishment of an appropriate indicator system. Overall, contemporary drip water that responds rapidly to precipitation is not suitable for the interpretation of paleoenvironmental information in karst rocky desertification regions reflected by the indicators in speleothems.

## 5. Conclusions

The hydrogeochemistry of drip water in the Shijiangjun Cave is controlled by the chemical dissolution of calcareous dolomite and shows the weak alkalinity of typical karst water. Both calcium and magnesium ions are dominant in the cations, and bicarbonate is dominant in the anions. All hydrochemical types of the selected three drip waters are classified as the HCO_3_-Ca·Mg type. According to the dynamic variations in the hydrogeochemistry of drip water over time, it is concluded that the response of W1 to precipitation was more delayed than those of W2 and W3; thus the three drip sites are grouped into two categories, including the delayed (W1) and rapid response types (W2 and W3), which resulted from the influence of different hydrogeochemical processes during the investigation. Specifically, the chemical compositions of W1 are constrained by the “piston effect”, and those of W2 and W3 are significantly affected by the rainwater dilution effect owing to the well-connected seepage flow paths. The relatively lower Ca^2+^ concentrations in the drip water of the Shijiangjun Cave during the hydrological year could be related to the effect of prior calcite precipitation, which needs to be verified by conducting a continuous and scientific observation in our following monitoring.In addition to the bedrock dissolution, the soil layer is one of the essential element sources for drip water, and the response sensitivity of the hydrogeochemical indicators for drip water to the external atmospheric precipitation is restricted by the karst rocky desertification degree during the rainy season. The influence of the overlying karst rocky desertification environment on the response of drip water to precipitation in the rainy season could be summarized as follows: the lower the degree of the karst rocky desertification, the more the response of the drip water is delayed; the higher degree of the karst rocky desertification, the faster the drip water responds to precipitation. This requires selecting more drip sites and conducting long-term and high-frequency observation for verification in the future. For the Shijiangjun Cave, several kinds of parameters, such as sulfate and nitrate in the drip waters of W2 and W3, have probably inherited the signals of the overlying soil environment and have the potential of indicating the degree of surface karst rocky desertification depending on the drip water hydrogeochemistry. However, a more accurate conclusion must be achieved on the basis of the stable isotope determination of nitrogen or sulfur in further exploration. Ultimately, it is worth noting that the selections of the drip sites and indicators in revealing the evolutional history of karst rocky desertification using speleothems should be paid more attention.

## Figures and Tables

**Table 1:** Temporal-ordered parameters and descriptive statistics of the drip water hydrogeochemical composition in the Shijiangjun Cave.

W1 (n = 18)	Drip Rate /mL·min^−1^	pH	EC /μS·cm^−1^	HCO_3_^−^ /mg·L^−1^	SO_4_^2−^ /mg·L^−1^	NO_3_^−^ /mg·L^−1^	Cl^−^ /mg·L^−1^	K^+^ /mg·L^−1^	Na^+^ /mg·L^−1^	Ca^2+^ /mg·L^−1^	Mg^2+^ /mg·L^−1^	Sr^2+^ /mg·L^−1^	SI_C_	SI_D_
	2.21	8.41	360	213.5	21.56	3.62	3.55	0.95	0.59	40.22	20.97	0.016	0.72	1.34
	2.1	8.41	370	213.5	21.28	3.55	5	1.5	0.59	41	21.1	0.016	0.73	1.35
	2.05	8.41	384	213.5	21.01	3.52	7.09	2.28	0.58	41.64	21.27	0.016	0.74	1.36
	2.03	8.49	354	213.5	21.34	3.54	3.55	0.58	1.12	43.17	21.32	0.019	0.83	1.53
	2.02	8.52	442	207.4	20.7	3.12	14.18	7.32	0.58	39.64	20.97	0.016	0.81	1.52
	2.16	8.78	364	207.4	20.14	3.38	5.67	1.22	0.59	38.53	20.79	0.016	1.03	1.96
	2.04	8.78	362	207.4	20.3	3.2	5.3	1.1	0.57	39	20.7	0.016	1.03	1.96
	2.01	8.78	361	213.5	20.5	3.19	4.96	1.04	0.56	39.5	20.65	0.016	1.05	1.99
	1.99	8.78	351	201.3	20.21	3.16	3.55	0.39	0.57	39.57	20.8	0.015	1.03	1.96
	1.89	8.54	348	201.3	20.62	3.1	3.55	0.4	0.75	39.71	20.73	0.016	0.82	1.53
	2.04	8.54	353	201.3	20.73	3.02	3.55	0.37	0.58	40.08	20.99	0.016	0.82	1.53
	2.24	8.74	357	183	20.05	2.94	3.55	0.34	0.54	40.11	20.93	0.016	0.96	1.81
	2.1	8.7	355	170.8	20.1	2.85	3.55	0.4	0.7	41	21	0.017	0.91	1.7
	1.85	8.61	354	170.8	20.13	2.84	3.55	0.43	0.78	41.28	21.28	0.017	0.83	1.55
	1.83	8.76	351	213.5	20.28	2.84	3.55	0.53	0.98	40.22	21.29	0.016	1.04	1.99
	1.95	8.57	360	219.6	20.89	2.47	7.8	3.05	0.63	40.02	21.25	0.016	0.88	1.66
	1.84	8.65	355	225.7	21.28	2.77	4.96	1.35	1.05	40.46	21.27	0.017	0.97	1.84
	1.91	8.67	381	170.8	21.27	2.52	12.05	5.87	0.94	41.84	22.09	0.017	0.89	1.67
Min	1.83	8.41	348	170.8	20.05	2.47	3.55	0.34	0.54	38.53	20.65	0.02	0.72	1.34
Max	2.24	8.78	442	225.7	21.56	3.62	14.18	7.32	1.12	43.17	22.09	0.02	1.05	1.99
Average	2.01	8.62	364.56	202.66	20.69	3.09	5.5	1.62	0.71	40.39	21.08	0.02	0.89	1.68
S.D	0.12	0.14	21.66	17.2	0.5	0.34	3.08	1.97	0.19	1.12	0.34	0.0008	0.11	0.23
**W2** **(n = 18)**	**Drip Rate** **/mL·min^−1^**	**pH**	**EC** **/μS·cm** **^−^** **^1^**	**HCO_3_** **^−^** **/mg** **·** **L** **^−^** **^1^**	**SO_4_^2^** **^−^** **/mg** **·** **L** **^−^** **^1^**	**NO_3_** **^−^** **/mg** **·** **L** **^−^** **^1^**	**Cl** **^−^** **/mg** **·** **L** **^−^** **^1^**	**K^+^** **/mg** **·** **L** **^−^** **^1^**	**Na^+^** **/mg** **·** **L** **^−^** **^1^**	**Ca^2+^** **/mg** **·** **L** **^−^** **^1^**	**Mg^2+^** **/mg** **·** **L** **^−^** **^1^**	**Sr^2+^** **/mg** **·** **L** **^−^** **^1^**	**SI_C_**	**SI_D_**
	2.03	8.66	274	158.6	15.67	2.8	3.55	0.23	0.51	22.19	19.43	0.008	0.6	1.31
	1.95	8.7	275	170.8	15.4	2.76	2.13	0.23	0.52	22.7	19.7	0.008	0.67	1.46
	1.8	8.74	275	170.8	15.05	2.75	1.42	0.22	0.53	22.92	19.89	0.008	0.71	1.54
	1.75	8.68	272	152.5	16.21	2.72	4.25	0.32	0.64	23.72	19.96	0.009	0.63	1.36
	1.82	8.78	272	170.8	14.91	2.7	3.55	0.21	0.53	23.43	19.82	0.008	0.76	1.62
	1.65	8.82	273	170.8	15.07	2.74	4.25	0.24	0.54	22.42	19.72	0.008	0.77	1.67
	1.59	8.8	274	176.9	15.07	2.8	3.55	0.21	0.53	23	19.8	0.008	0.78	1.67
	1.45	8.78	274	176.9	15.1	2.85	2.84	0.2	0.52	23.29	19.9	0.008	0.77	1.65
	1.41	8.85	271	170.8	15.91	2.72	2.13	0.22	0.56	23.06	19.82	0.008	0.81	1.73
	1.38	8.7	270	176.9	14.98	2.7	4.25	0.2	0.53	23.62	20.04	0.008	0.7	1.51
	1.71	8.7	273	158.6	14.94	2.64	2.13	0.2	0.53	23.38	19.89	0.008	0.66	1.42
	2.1	8.75	274	183	15.04	2.65	2.84	0.22	0.57	23.61	19.86	0.009	0.76	1.62
	2.01	8.7	272	176.9	16	2.68	2.84	0.21	0.54	23.5	19.8	0.008	0.7	1.5
	2.02	8.7	270	176.9	16.49	2.71	3.55	0.21	0.53	23.26	19.73	0.008	0.7	1.5
	2.03	8.77	271	170.8	15.39	2.69	2.84	0.19	0.66	23.67	20.13	0.009	0.75	1.61
	2.09	8.73	272	170.8	16.38	2.7	2.13	0.21	0.54	23.63	20.11	0.008	0.71	1.54
	1.98	8.74	273	176.9	17.26	2.73	2.13	0.2	0.54	23.57	19.85	0.009	0.74	1.58
	1.43	8.78	272	176.9	16.88	2.71	2.84	0.27	1.06	24.83	19.9	0.01	0.79	1.67
Min	1.38	8.66	270	152.5	14.91	2.64	1.42	0.19	0.51	22.19	19.43	0.01	0.6	1.31
Max	2.1	8.85	275	183	17.26	2.85	4.25	0.32	1.06	24.83	20.13	0.01	0.81	1.73
Average	1.79	8.74	272.61	171.48	15.65	2.72	2.95	0.22	0.58	23.32	19.85	0.01	0.72	1.55
S.D	0.25	0.05	1.54	7.8	0.74	0.05	0.85	0.03	0.13	0.58	0.16	0.0006	0.06	0.12
**W3** **(n = 18)**	**Drip Rate** **/mL·min^−1^**	**pH**	**EC** **/μS·cm** **^−^** **^1^**	**HCO_3_** **^−^** **/mg** **·** **L** **^−^** **^1^**	**SO_4_^2^** **^−^** **/mg** **·** **L** **^−^** **^1^**	**NO_3_** **^−^** **/mg** **·** **L** **^−^** **^1^**	**Cl** **^−^** **/mg** **·** **L** **^−^** **^1^**	**K^+^** **/mg** **·** **L** **^−^** **^1^**	**Na^+^** **/mg** **·** **L** **^−^** **^1^**	**Ca^2+^** **/mg** **·** **L** **^−^** **^1^**	**Mg^2+^** **/mg** **·** **L** **^−^** **^1^**	**Sr^2+^** **/mg** **·** **L** **^−^** **^1^**	**SI_C_**	**SI_D_**
	2.37	8.7	232	170.8	5.8	1.91	3.55	0.23	0.31	16.86	19.28	0.006	0.56	1.35
	2.25	8.72	232	164.7	5.62	1.9	3	0.26	0.31	17	19.5	0.006	0.56	1.36
	2.18	8.75	232	158.6	5.59	1.9	2.84	0.29	0.32	17.04	19.57	0.006	0.58	1.39
	2	8.71	232	164.7	4.99	1.86	2.84	0.26	0.3	16.77	19.49	0.006	0.55	1.34
	1.72	8.86	232	207.4	6.33	1.88	3.55	0.23	0.33	16.78	19.24	0.006	0.77	1.78
	1.64	8.81	231	183	6.43	1.87	2.13	0.25	0.49	19.13	19.66	0.008	0.73	1.65
	1.87	8.8	232	183	6.1	1.81	2.83	0.22	0.35	17	19.4	0.007	0.67	1.59
	1.94	8.79	232	183	5.83	1.77	3.55	0.2	0.3	16.59	19.27	0.006	0.66	1.56
	2.11	8.84	230	164.7	5.9	1.81	2.84	0.21	0.59	16.84	19.15	0.007	0.67	1.56
	2.3	8.73	230	176.9	5.37	1.89	3.55	0.2	0.31	16.66	19.14	0.006	0.59	1.42
	2.51	8.74	231	183	6.05	1.78	2.84	0.21	0.3	16.79	19.38	0.006	0.62	1.47
	2.1	8.77	231	164.7	5.9	1.79	2.84	0.2	0.32	16.57	19.27	0.006	0.6	1.44
	2.32	8.74	232	176.9	6.1	1.79	3.2	0.29	0.4	17	19.7	0.007	0.61	1.46
	2.55	8.74	232	176.9	6.27	1.8	3.55	0.37	0.52	19.15	19.8	0.008	0.66	1.51
	2.61	8.78	230	176.9	5.84	1.86	2.84	0.21	0.3	16.84	19.56	0.006	0.64	1.52
	2.83	8.76	231	176.9	6.67	1.8	3.55	0.22	0.31	16.76	19.35	0.006	0.62	1.48
	2.21	8.75	232	158.6	6.21	1.81	2.13	0.22	0.49	17.44	19.6	0.007	0.59	1.4
	2.45	8.76	231	183	6.42	1.86	1.42	0.22	0.31	16.35	18.81	0.006	0.62	1.49
Min	1.64	8.7	230	158.6	4.99	1.77	1.42	0.2	0.3	16.35	18.81	0.01	0.55	1.34
Max	2.83	8.86	232	207.4	6.67	1.91	3.55	0.37	0.59	19.15	19.8	0.01	0.77	1.78
Average	2.22	8.76	231.39	175.21	5.97	1.84	2.94	0.24	0.36	17.09	19.4	0.01	0.63	1.49
S.D	0.31	0.04	0.78	11.8	0.41	0.05	0.59	0.04	0.09	0.78	0.24	0.0006	0.06	0.11

**Table 2:** Previous hydrochemical data compilation of rainwater during rainy seasons in the study area (unit: mg·L^−1^).

Date	pH	K^+^	Na^+^	Ca^2+^	Mg^2+^	Sr^2+^	HCO_3_^–^	SO_4_^2−^	NO_3_^−^	Cl^−^
12 May 2014	7.22	0.32	0.11	2.27	0.6	0.003	8.7	1.4	0.88	0.12
17 June 2014	7.13	0.25	0.13	2.8	0.54	0.002	10.4	1.48	0.45	0.3
9 August 2014	7.35	0.37	0.2	2.41	0.63	0.003	12.2	1.33	0.9	0.38
15 June 2015	7.36	0.33	0.18	2.6	0.44	0.002	11.3	0.89	1.01	0.31
14 July 2015	7.41	0.28	0.15	1.83	0.5	0.003	11.2	1.42	0.64	0.22

**Table 3:** Comparison of the drip water response time to precipitation among different grades of karst rocky desertification regions in the rainy season.

Cave	Sampling Period	Location	Vegetation Type	Soil Thickness/cm	Karst Desertification Grade	Response Time	References
Shijiangjun	2016	Anshun, Guizhou	Shrub, grass	5~46	Moderate	38~42 h	This study
Qixing	2003	Duyun, Guizhou	Shrub	50~90	Mild	27~40 d	[20]
Liangfeng	2003	Libo, Guizhou	Primordial forest	100	None	36 d	[20]
Xiniu	2003	Zhenning, Guizhou	Shrub grass	20~60	Mild	28 d	[20]
Xiaoyan	2014	Guilin, Guangxi	Shrub grass	30~100	Moderate-intense	<48 h	[24,25]
Furong	2010	Wulong, Chongqing	Arbor, shrub	>30	None	>30 d	[39]
Xueyu	2012~2013	Fengdu, Chongqing	Arbor, shrub	0~50	None	30 d	[47]
Panlong	2012	Guilin, Guangxi	Shrub	30~100	Mild	15 d	[48]

## Data Availability

The data presented in this study are available on request from the corresponding author.
